# Chromatin folding and DNA replication inhibition mediated by a highly antitumor-active tetrazolato-bridged dinuclear platinum(II) complex

**DOI:** 10.1038/srep24712

**Published:** 2016-04-20

**Authors:** Ryosuke Imai, Seiji Komeda, Mari Shimura, Sachiko Tamura, Satoshi Matsuyama, Kohei Nishimura, Ryan Rogge, Akihiro Matsunaga, Ichiro Hiratani, Hideaki Takata, Masako Uemura, Yutaka Iida, Yuko Yoshikawa, Jeffrey C. Hansen, Kazuto Yamauchi, Masato T. Kanemaki, Kazuhiro Maeshima

**Affiliations:** 1 Biological Macromolecules Laboratory, Structural Biology Center, National Institute of Genetics, Mishima, Shizuoka 411-8540, Japan; 2 Department of Genetics, School of Life Science, Sokendai (Graduate University for Advanced Studies), Mishima, Shizuoka 411-8540, Japan; 3 Faculty of Pharmaceutical Sciences, Suzuka University of Medical Science, Suzuka, Mie, 513-8670; 4 CREST, JST, 4-1-8 Honcho, Kawaguchi, Saitama 332-0012, Japan; 5 Department of Intractable Diseases, Research Institute, National Center for Global Health and Medicine, Shinjuku, Tokyo 162-8655, Japan; 6 RIKEN SPring-8 Center, 1-1-1 Kouto, Sayo-cho, Sayo-gun, Hyogo 679-5148, Japan; 7 Department of Precision Science & Technology, Graduate School of Engineering, Osaka University, 2-1 Yamada-oka Suita, Osaka, Japan 565-0871; 8 Center for Frontier Research, National Institute of Genetics, Yata 1111, Mishima, Shizuoka 411-8540, Japan; 9 Department of Biochemistry and Molecular Biology, Colorado State University, Fort Collins, CO 80523, USA; 10 Frontier Research Base for Global Young Researchers, Graduate School of Engineering Osaka University, 2-1 Yamada-oka, Suita, Osaka 565-0871, Japan; 11 Inorganic Analysis Laboratories, Toray Research Center, Inc., 3-3-7, Sonoyama, Otsu, Shiga 520-8567, Japan; 12 Research Organization of Science and Engineering, Ritsumeikan University, Kusatsu, Shiga 525-8577, Japan; 13 PRESTO, JST, 4-1-8 Honcho, Kawaguchi, Saitama 332-0012, Japan; 14 Laboratory for Developmental Epigenetics, RIKEN Center for Developmental Biology, Kobe 650-0047, Japan

## Abstract

Chromatin DNA must be read out for various cellular functions, and copied for the next cell division. These processes are targets of many anticancer agents. Platinum-based drugs, such as cisplatin, have been used extensively in cancer chemotherapy. The drug–DNA interaction causes DNA crosslinks and subsequent cytotoxicity. Recently, it was reported that an azolato-bridged dinuclear platinum(II) complex, 5-H-Y, exhibits a different anticancer spectrum from cisplatin. Here, using an interdisciplinary approach, we reveal that the cytotoxic mechanism of 5-H-Y is distinct from that of cisplatin. 5-H-Y inhibits DNA replication and also RNA transcription, arresting cells in the S/G2 phase, and are effective against cisplatin-resistant cancer cells. Moreover, it causes much less DNA crosslinking than cisplatin, and induces chromatin folding. 5-H-Y will expand the clinical applications for the treatment of chemotherapy-insensitive cancers.

The long strands of genomic DNA are wrapped around histone proteins and organized in cells as chromatin^1^. This chromatin DNA must be read out for various cellular functions, and copied for the next cell division (DNA replication) while maintaining integrity^1^. DNA transactions such as DNA replication/ repair/ recombination and RNA transcription are essential for cell viability and are targets of many anticancer agents currently used in cancer chemotherapy^2^.

Platinum-based drugs are the most commonly used anticancer agents, especially for the treatment of testicular, ovarian, and colorectal cancers. *Cis*-diamminedichloridoplatinum(II) (cisplatin, Fig. 1A)^3^,^4^ is a platinum-based DNA crosslinking agent that first proved the importance of platinum-DNA interactions^5^. Cisplatin and the other platinum-based drugs, such as carboplatin and oxaliplatin^6^, are considered to work in a similar fashion^7^. The platinum–DNA interactions make both inter- and intrastrand crosslinks in DNA, suppressing DNA replication^7^ and also RNA transcription^8^. For DNA replication process, intra-strand DNA crosslinks can be bypassed by some translesion synthesis (TLS) polymerases^7^. To deal with interstrand DNA crosslinks (ICLs), mammalian cells have evolved the Fanconi anemia (FA)/BRCA pathway, which is coupled with DNA replication^9^. FA is a rare genetic disorder characterized by progressive bone marrow failure and a highly elevated risk of hematological and squamous cancers^10^. To date, 19 FANC genes have been identified from FA patients, whose cells are highly sensitive to ICL-inducing agents, including cisplatin. Although the precise mechanism of ICL repair by the FA/BRCA pathway is not yet fully understood, it is clear that complex actions of FA proteins, nucleases, TLS polymerases, and homologous recombination proteins are involved. Importantly, loss of any protein involved in the FA/BRCA pathway ultimately leads to hypersensitivity to cisplatin.

A common problem with cisplatin and its derivatives is that prolonged treatment generates resistant cancer cells (e.g. ref. 11). Thus, it is important to develop new drugs that can kill cisplatin-resistant cancer cells. Ideally, a next-generation platinum-based drug should show high therapeutic efficiency and a wide anticancer spectrum, that is, a cytotoxicity profile with many cancer cell lines. Such a new drug is also likely to be effective against chemotherapy-insensitive cancers, such as pancreatic cancer. However, conventional platinum-based drugs usually have similar anticancer spectra and the clinical platinum-based drugs show cross-resistance^12^,^13^. Thus, a significant structural modification appears to be required to design candidate next-generation, platinum-based drugs^14^,^15^,^16^,^17^,^18^.

We previously introduced a series of cationic azolato-bridged dinuclear platinum(II) complexes [{*cis*-Pt(NH_3_)_2_}_2_(μ-OH)(μ-azolato)]^2+^ (azolato-bridged complexes), which have different structures from the conventional platinum-based drugs and overcome cross-resistance to cisplatin^19^,^20^,^21^. The azolato-bridged complexes possess a +2 ionic charge (e.g. Fig. 1A) and are known to interact with DNA both covalently and non-covalently. The covalent interaction provides bifunctional DNA adducts, such as 1,2-intrastrand crosslink with a minimal kink in the DNA^22^,^23^,^24^, which seems to be difficult to be repaired^25^. Via non-covalent interactions, the azolato-bridged complexes induce a conformational change in DNA structure^26^, presumably because of their cationic feature and slow rate of formation of covalent DNA crosslinks^27^,^28^.

The anticancer spectra of these azolato-bridged complexes, based on a panel of 39 human cancer cell lines (JFCR39), differed markedly from those of the conventional platinum-based drugs^21^. Thus, their mechanisms of action are also likely to differ. Among the azolato-bridged complexes, [{*cis*-Pt(NH_3_)_2_}_2_(μ-OH) (μ-tetrazolato-*N2*,*N3*)]^2+^ (5-H-Y) is the most promising anticancer drug candidate (Fig. 1A), and exhibits strikingly high *in vivo* antitumor efficacy against xenografted pancreatic cancer in nude mice, inhibiting tumor growth by 99% versus untreated controls^20^.

In the present study, using combined techniques of cell biology, structural biology, and biophysics, we investigated the cytotoxic mechanism of 5-H-Y. We found that the compound inhibits DNA replication and RNA transcription, and arrests treated cells in the S/G2 phase, causing great cytotoxicity. 5-H-Y has much less DNA crosslinking ability than cisplatin, and binds to DNA very tightly, inducing chromatin folding. We also found that DNA damage by 5-H-Y is repaired differently from ICL generated by cisplatin, and 5-H-Y is effective against cisplatin-resistant cancer cells. Our study provides a mechanistic insight into the cytotoxicity of 5-H-Y.

## Results

### The novel platinum complex 5-H-Y inhibits cell proliferation

**Figure 1:**
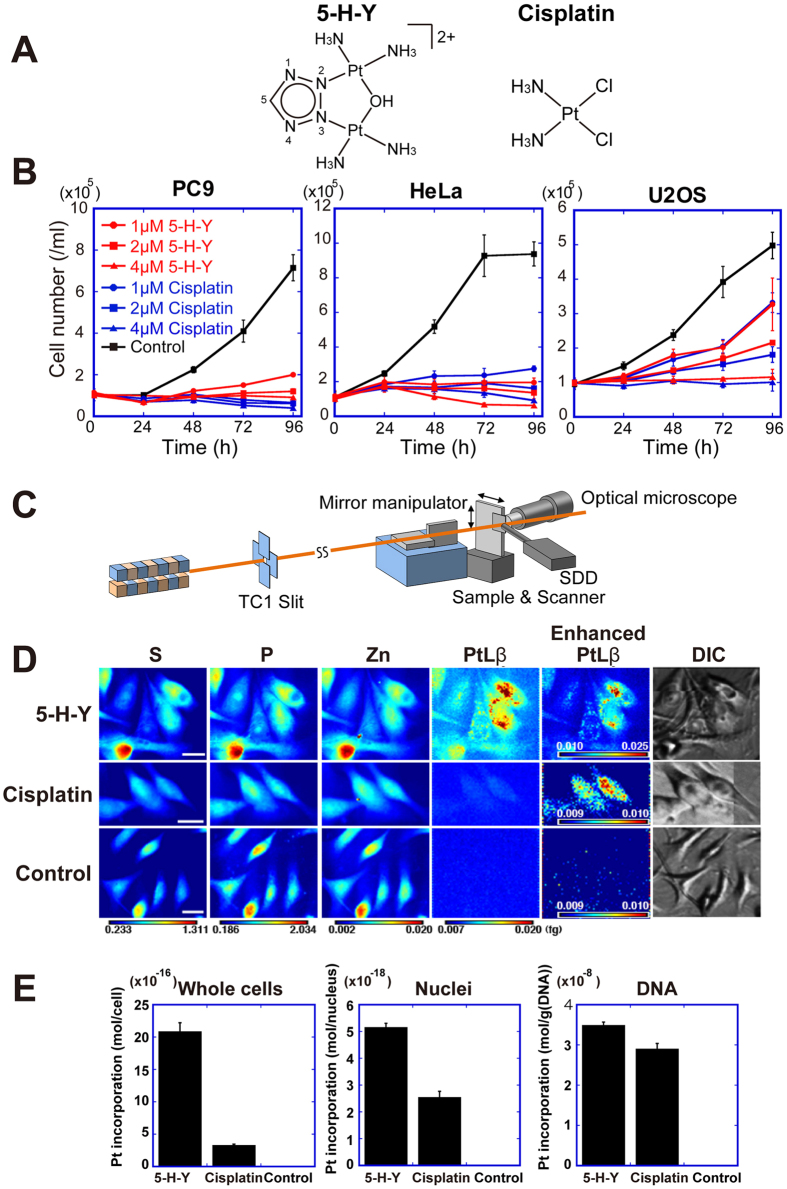
5-H-Y inhibits cell proliferation and is incorporated into cell nuclei. (**A**) Chemical structures of [{*cis-*Pt(NH_3_)_2_}_2_(μ-OH)(μ-tetrazolato-*N2*,*N3*)]^2+^ (5-H-Y) (left) and *cis*-diamminedichloridoplatinum(II) (cisplatin) (right). (**B**) Cell proliferation assays with 5-H-Y or cisplatin treatment. Four human cell lines (PC9, HeLa, U2OS, and TIG-1) were treated with the indicated concentrations of 5-H-Y or cisplatin, and the cell numbers were monitored from 0 to 96 h for human cells (for TIG-1, see also Fig. S1A). (**C**) Schematic view of scanning X-ray fluorescence microscopy. The X-ray beam, highly focused by a set of mirrors (KB-mirror) was focused on the cells refs 54 and 55. Then X-ray fluorescence was detected by the silicon drift detector (SDD). **(D)** SXFM analysis after drug treatment. Cell morphologies obtained by Nomarski (DIC). Brighter colors indicate a higher signal intensity of each element. Representative results are shown. Results are shown for 5-H-Y (top) and cisplatin (middle), untreated control PC9 cells (bottom). Note the high intensity of Pt in 5-H-Y treated cells. Pt, platinum signal, P, phosphorus, S, sulfur, Zn, zinc. Color bars indicate elemental content, expressed in fg/μm^2^. The phosphorus- and zinc -rich regions in the cells seem to be nuclei. Bars show 10 μm. (**E**) Amounts of platinum in PC9 whole cells, nuclei, and DNA fractions of 5-H-Y- and cisplatin-treated cells.

To evaluate the effects of 5-H-Y and cisplatin on cell growth inhibition, we first performed cell proliferation assays using four human cell lines (PC9, HeLa, U2OS, and TIG-1) (Figs 1B and S1A). PC9, HeLa, and U2OS cells are cancer cell lines and TIG-1 is a ‘normal’ human fibroblast line. Cell numbers were examined over time under various concentrations of 5-H-Y and cisplatin, from 0 to 96 h. Both drugs inhibited the growth of all cell lines tested in a similar manner (Figs 1B and S1A), consistent with a previous report^21^. These results suggest that 5-H-Y and cisplatin show comparable inhibitory effects on the proliferation of these cell lines.

### 5-H-Y is incorporated into cell nuclei

To gain clues into the mechanism of 5-H-Y cytotoxicity, we next investigated the intracellular localization of 5-H-Y and cisplatin in PC9 cells. It is normally not possible to examine drug localization by conventional cell biological methods. For this purpose, we used scanning X-ray fluorescence microscopy (SXFM)^29^,^30^ (Fig. 1C). This method enables the detection of the target elements at a single-cell level and gives a cellular localization profile of these elements. We examined various element localizations in both 5-H-Y- and cisplatin-treated PC9 cells. We detected many elements, including phosphorus, sulfur, zinc, and platinum (Fig. 1D). Signals of phosphorus, sulfur, and zinc mainly reflect on localizations of nucleic acids, proteins, and DNA-binding proteins, respectively^29^,^30^. In 5-H-Y-treated cells, platinum was observed throughout the cells, including in the nuclei. The cisplatin-treated cells also showed platinum signals, consistent with our previous study^29^,^30^,^31^.

To further confirm these findings, we fractionated the drug-treated PC9 cells as whole cells, nuclei (detergent-treated), and naked DNA fractions, and analyzed the amount of platinum in each by inductively coupled plasma-mass spectrometry (ICP-MS). Considerable amounts of platinum were present in all fractions from both cell groups (Fig. 1E), suggesting that 5-H-Y and cisplatin are incorporated into nuclei and some of the drug interacts tightly with DNA. Because 5-H-Y was detected in nuclei and found even in the DNA fraction, similar to the case of cisplatin, we next paid attention to DNA replication and RNA transcription.

### 5-H-Y inhibits DNA replication and arrests the cell cycle in the S/G2 phase

**Figure 2:**
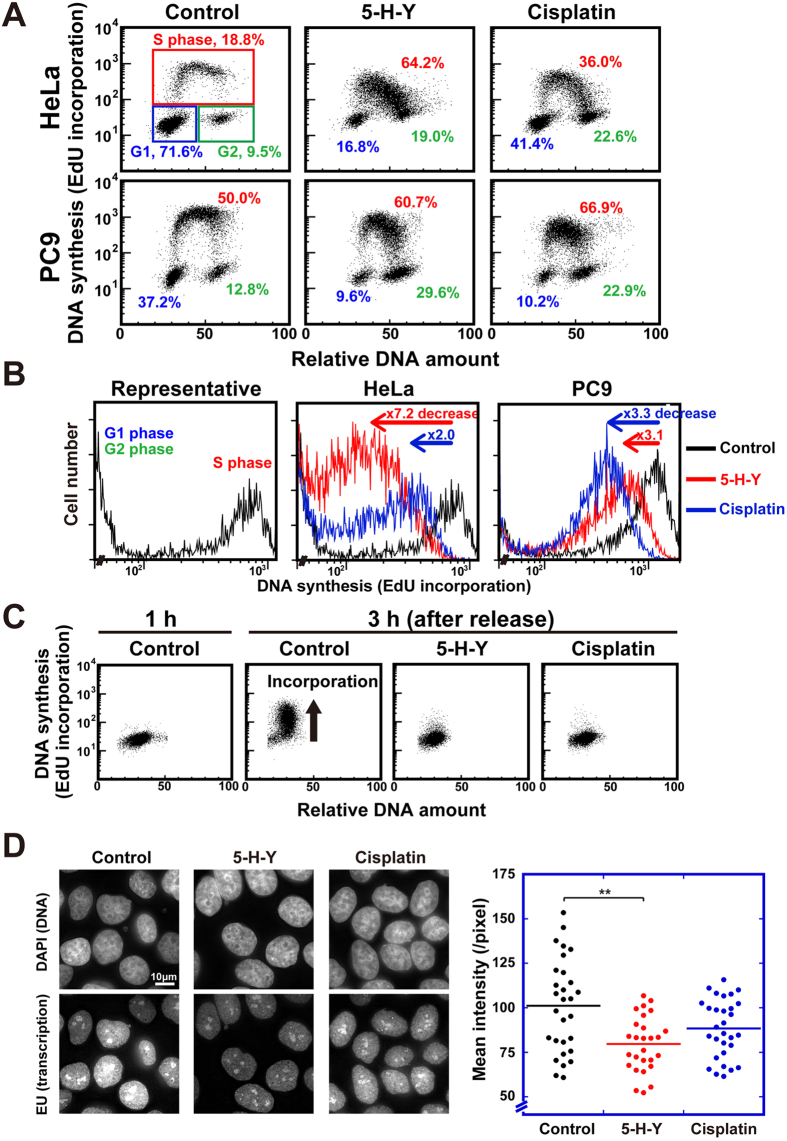
5-H-Y inhibits DNA replication and RNA transcription, arresting the cells in the S/G2 phase. (**A**) Flow cytometry results for HeLa cells (upper row) and PC9 cells (lower row) with/without 5-H-Y (2 μM) or cisplatin (2 μM). Vertical and horizontal axes show DNA synthesis activity (EdU incorporation) and DNA amount, respectively. Each dot represents a single cell and results using 10,000 cells are plotted. In the plot of control HeLa, the corresponding cell cycle stages are indicated. Percentages of each cell cycle population are indicated. See also Fig. S2. (**B**) EdU incorporation versus cell numbers plots of (**A**). Left panel shows a representative plot (control HeLa). Note that EdU incorporation was high in the S-phase. Fold decreases in EdU incorporation upon 5-H-Y (red) or cisplatin (blue) treatment are indicated in the plots of HeLa (middle) and PC9 (right). Note the several-fold decreases in EdU incorporation in the 5-H-Y (red) or cisplatin (blue) treated cells. (**C**) Results for HeLa cells synchronized at G1/S by nocodazole-thymidine block with/without 5-H-Y (2 μM) or cisplatin (2 μM). 5-H-Y and cisplatin both inhibit very early phases of DNA replication. (**D**) Effect of 5-H-Y on RNA transcription *in vivo*. (Left) Fluorescence microscopy images of 5-H-Y or cisplatin-treated cells. DNA stain, upper; EU fluorescent labeling, lower. (Right) Dot plot of the mean intensity of EU fluorescence in each nucleus (each group, n = 27–30). **p < 0.01, Student’s t-test.

Cisplatin binds covalently to DNA, which trigger inhibition of DNA replication, causing cell cycle arrest in the S/G2 phase^32^. To examine the effects of 5-H-Y on the cell cycle, we monitored the cell cycle stages of drug-treated (24 h) HeLa, U2OS, PC9, and TIG-1 cells, using flow cytometry (FCM) (Figs 2A,B and S2). Cisplatin inhibited the incorporation of a thymidine analog, 5-ethynyl-2′-deoxyuridine (EdU), into newly synthesized DNA, suggesting that DNA replication was inhibited (Figs 2A,B and S2). Consistently, cisplatin-treated cells were arrested in the S/G2 phase (Figs 2A and S2A). In 5-H-Y-treated cells, a 3- to 10-fold reduction of EdU incorporation and cell cycle arrest in S/G2 were observed, similar to the effects of cisplatin (Figs 2A and S2A). Furthermore, we synchronized HeLa cells at the G1/S phase boundary before treatment with 5-H-Y for 15 h (Experimental scheme is shown in Fig. S3). EdU incorporation was almost completely inhibited after release from the G1/S block (Fig. 2C). These results suggest that 5-H-Y has an inhibitory effect on DNA replication, as does cisplatin.

While we observed a similar inhibition of DNA replication between the cells treated with 5-H-Y and cisplatin for 15 h (Fig. 2C) or 24 h (Fig. 2A,B), we found that 5-H-Y has a more rapid effect on DNA replication than cisplatin (Fig. S4). When we treated HeLa cells with the drugs for 2 h, 5-H-Y inhibited DNA replication more severely than cisplatin (Fig. S4). This effect was also observed in other human cell lines (Fig. S4B).

### 5-H-Y reduces RNA transcription

Since it was reported that cisplatin could inhibit RNA transcription e.g. ref. 8, we examined effect of 5-H-Y on RNA transcription by incorporation of 5-Ethynyl uridine (EU). 5-H-Y treatment decreased the EU incorporation into newly synthesized RNA in the cells (Fig. 2D), suggesting that global RNA transcription was reduced in the treated cells. Consistent with the previous reports, the EU incorporation in cisplatin-treated cells was also reduced (Fig. 2D).

### 5-H-Y induces fewer γH2AX foci than cisplatin

**Figure 3:**
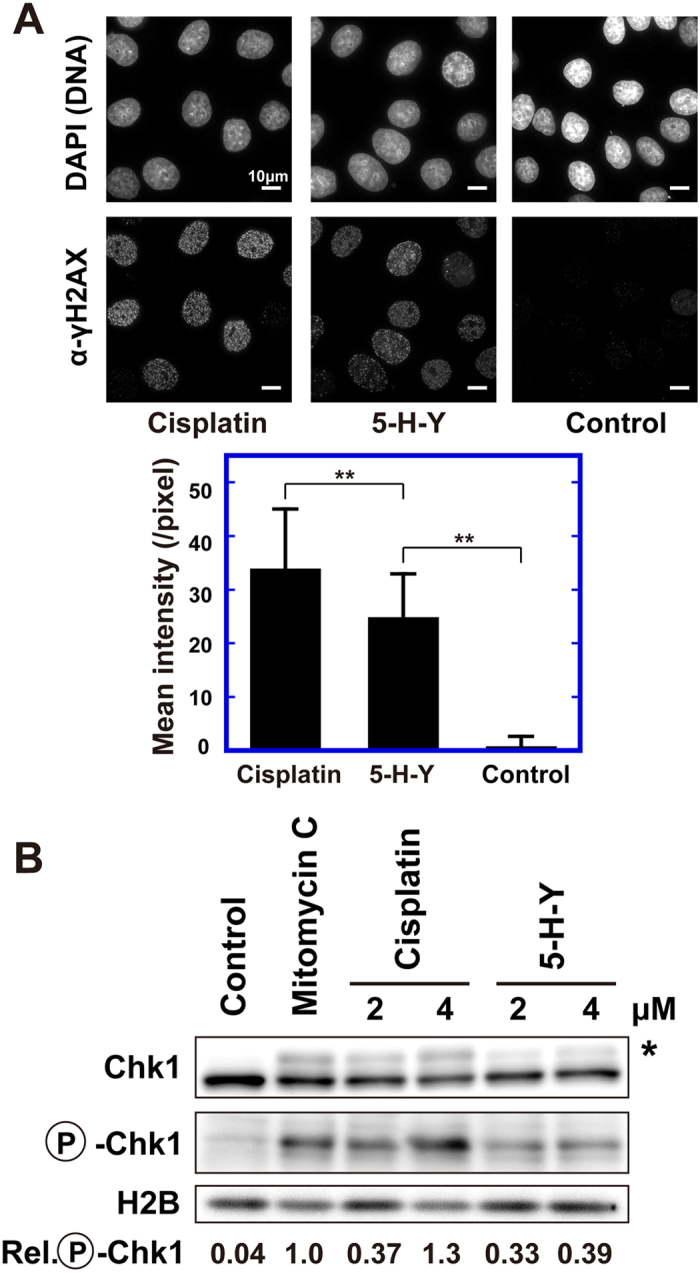
DNA damage response in 5-H-Y treated cells. (**A**) γH2AX foci formation in 5-H-Y or cisplatin-treated HeLa cells. DNA stain, upper; anti-γH2AX antibody staining, lower. Scale bars are 10 μm. The bar graph indicates quantification of the γH2AX signal intensity averaged from ~50 nuclei. Note that the signal in 5-H-Y-treated cells was significantly lower than in cisplatin-treated cells. **p < 0.01, Student’s t-test. (**B**) Chk1 activation on drug treatment. Western blotting analysis of cell lysates using anti-Chk1 (1^st^ row) and anti-phospho-Chk1 (P-Chk1) (2^nd^ row) antibody. In the 1^st^ row, the position of phosphorylated (activated) Chk1 is marked by the asterisk. Control, no treatment; mitomycin C, mitomycin C treatment for efficient DNA crosslinking. The third row is a loading control using H2B. The values at the bottom indicate quantification of the phosphorylated Chk1 signal intensity. Note that the relative intensity of phosphorylated signal in 5-H-Y-treated cells was considerably lower than that in cisplatin-treated cells. The blots were cropped at the positions of the proteins for clarity and space considerations.

Next, we examined foci formation of phospho-H2AX (γH2AX) in the 5-H-Y-treated cells, which are often associated with DNA double-strand breaks (DSBs)^33^,^34^ (Fig. 3A). We observed γH2AX foci in various 5-H-Y-treated cells, such as HeLa, PC9, and TIG-1 cells, but the foci were significantly fewer and weaker than those observed in cisplatin-treated cells (Figs 3A and S5). In addition, in PC9 cells, the γH2AX-foci localization seemed to differ between 5-H-Y- and cisplatin-treated cells: the foci with cisplatin were enriched in the nuclear rim, while those with 5-H-Y were localized more uniformly in the nucleoplasm (Fig. S5). Furthermore, when we examined checkpoint activation by hyperphosphorylation of the checkpoint mediator Chk1 in the 5-H-Y treated cells, significantly lower levels of Chk1 phosphorylation were seen than in cisplatin- or mitomycin C-treated cells (Fig. 3B). These results suggest that DNA damages induced by 5-H-Y are somehow distinct from those by cisplatin.

### 5-H-Y provides less amount of DNA crosslinks

**Figure 4:**
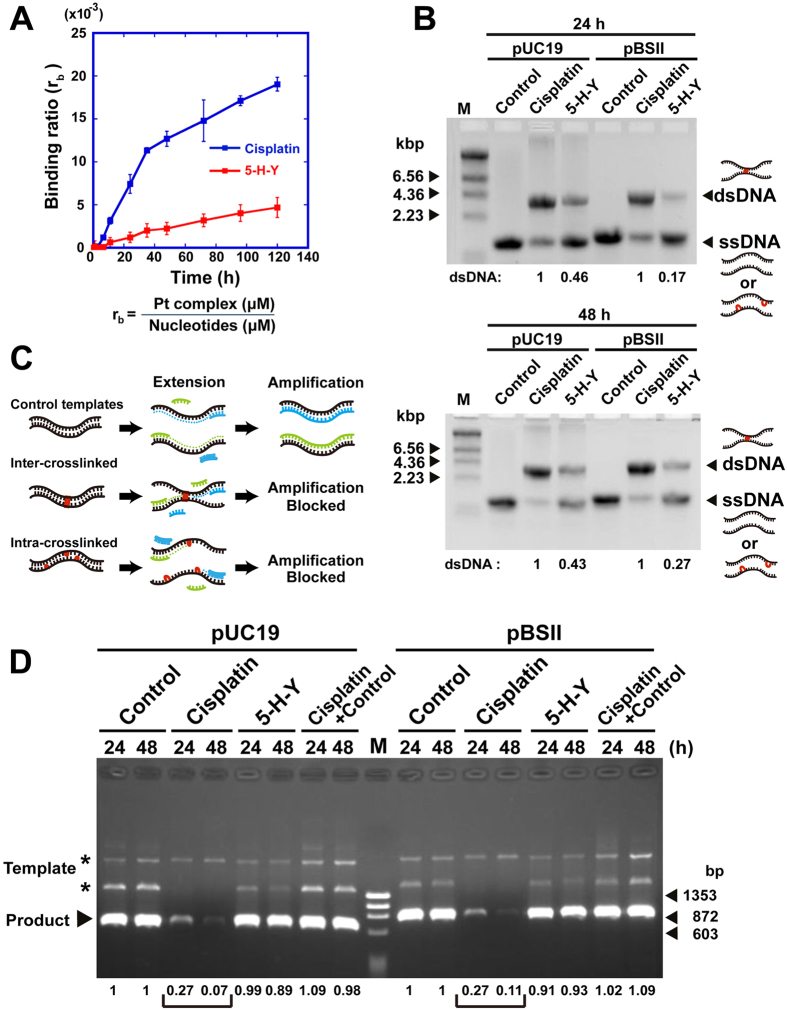
Much lower interstrand crosslinking (ICL) activity of 5-H-Y. (**A**) Covalent binding of cisplatin (blue square) and 5-H-Y (red square) to calf-thymus DNA (*n* = 4). The r_b_ value is defined as the molar ratio of platinum complex bound per nucleotide. (**B**) Interstrand crosslinking of drug-treated plasmid DNA. Two types of plasmid DNAs, pUC19 (left) and pBluescript II (pBSII) (right), were treated with no drug (Control), cisplatin, or 5-H-Y for 24 h (upper) or 48 h (lower). The treated plasmid DNAs were electrophoresed on alkaline agarose gels. The gels with EtBr staining are shown. The positions of dsDNA, representing interstrand crosslinks, and ssDNA, including no crosslinks and intrastrand crosslinks, are shown. Values below the gels indicate intensities of dsDNA normalized by that of cisplatin. Note that there is much more dsDNA in cisplatin-treated DNA than in 5-H-Y-treated DNA. The two DNA templates, pUC19 and pBSII, produced similar results. (**C**) Experimental scheme of PCR amplification. DNA templates were treated with cisplatin or 5-H-Y. If inter-strand (middle) or intrastrand (bottom) crosslinks occur in the template DNA, DNA amplification by PCR is inhibited. (**D**) PCR results. Two types of plasmid DNAs, pUC19 (left) and pBluescript II (pBSII) (right), were used as PCR templates. They were treated with no drug (Control), cisplatin, or 5-H-Y for 24 h or 48 h. The PCR products (marked with arrow) on the agarose gel after electrophoresis are shown. Values below the gel indicate the fluorescent intensities of the PCR product normalized by that of control. In the “Cisplatin + Control” lanes, PCR was performed using mixed templates of cisplatin-treated and no-treated plasmids. Note that PCR using cisplatin-treated template produced much less product.

To investigate the DNA crosslinking ability of 5-H-Y, DNA purified from calf thymus was incubated with 5-H-Y or cisplatin for various periods of time. Quantification analysis showed that ~5-fold less 5-H-Y than cisplatin was bound covalently to DNA (Fig. 4A).

Next, we examined the frequency of interstrand DNA crosslink (ICL) formation directly using drug-treated plasmid DNAs (pUC19 and pBluescript II) separated in alkaline agarose gel (Fig. 4B). In both cisplatin-treated plasmid DNAs, there was much more dsDNA (representing ICLs; arrowheads in Fig. 4B) than in 5-H-Y-treated DNAs, suggesting that 5-H-Y induces 2.2- to 5.9-fold less ICL than cisplatin.

Furthermore, we performed semi-quantitative PCR using 5-H-Y or cisplatin-treated DNAs (pUC19 and pBluescript II) as the template (Fig. 4C). The plasmid DNAs were treated with 5-H-Y or cisplatin, purified and used as template DNAs for PCR. In the PCR with the cisplatin-treated template, ~10-fold less PCR products were detected than with 5-H-Y-treated one (Brackets in Figs 4D and S6). Because PCR using mixed DNA templates of cisplatin-treated and untreated plasmids showed successful amplification (the “Cisplatin + Control” lanes in Fig. 4D), DNA crosslinks, not DNA polymerase inhibition by cisplatin, suppressed the PCR reaction. The two DNA templates, pUC19 and pBluescript II, produced similar results, showing that the result had no DNA sequence dependency (Fig. 4D). We concluded that 5-H-Y generates intra- and interstrand crosslinks with much lower frequency compared to cisplatin.

### 5-H-Y binds tightly to chromatin DNA and folds chromatin *in vitro* and *in vivo*

**Figure 5:**
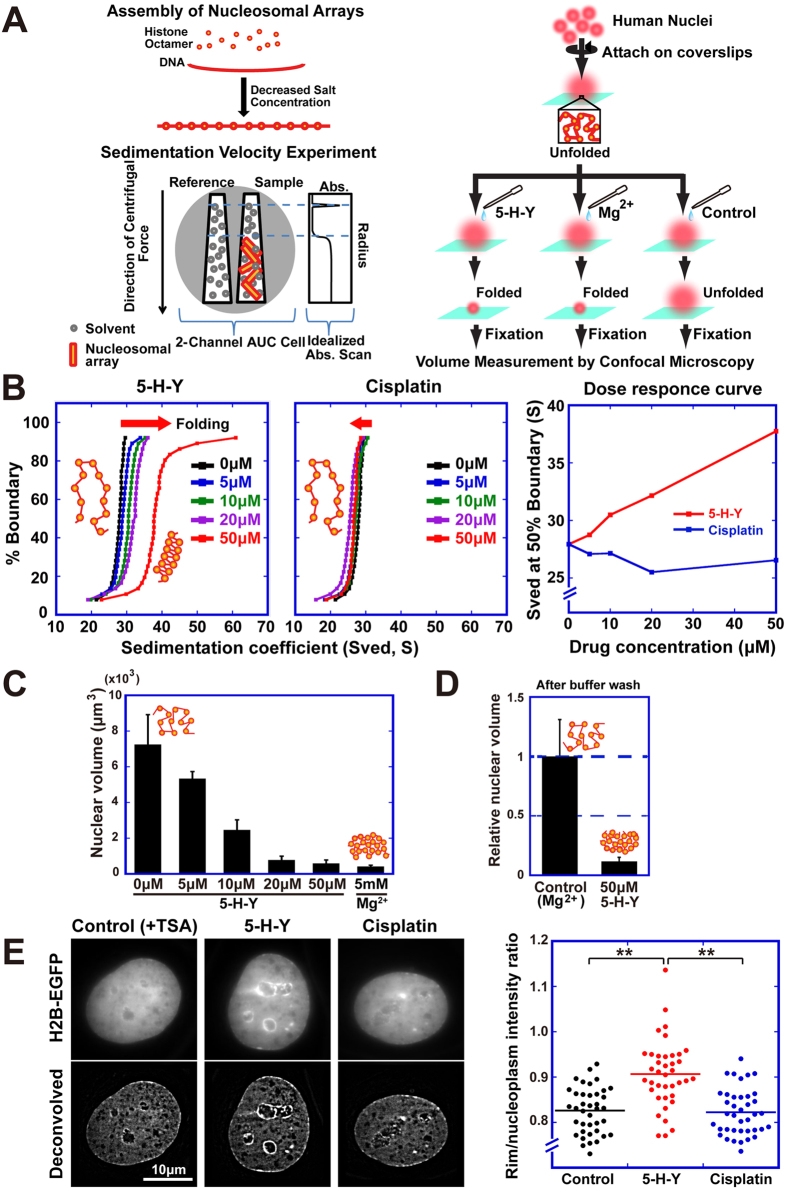
5-H-Y binds to chromatin DNA tightly and folds chromatin *in vitro* and *in vivo.* (**A**) Experimental scheme of the two chromatin folding assays: ultracentrifuge assay (left) and nuclear volume assay (right). (**B**) 5-H-Y induces chromatin folding. Samples of reconstituted nucleosome fibers were exposed to the indicated concentrations of 5-H-Y (left) or cisplatin (center) and analyzed by sedimentation velocity analytical ultracentrifugation. The integral distribution of diffusion-corrected sedimentation coefficients obtained after analysis of the data by the method of Demeler and van Holde are shown^60^,^61^. (Right) Summary of analytical ultracentrifuge-SV results. Values at the 50% boundary are displayed as a function of drug concentration added 5-H-Y (red) or cisplatin (blue). (**C**) Nuclear volume was decreased by 5-H-Y in a dose-dependent manner. Nuclei treated with 50 μM 5-H-Y showed a 12-fold decrease in the volume. This indicates that 5-H-Y induces chromatin folding. The nuclei treated with 5 mM Mg^2+^ were prepared as a control for the nuclei with highly folded chromatin. The error bars represent the standard deviation. For each point, *n* = ~100. (**D**) Volumes between Mg^2+^-pretreated and 5-H-Y-pretreated nuclei after buffer washing. When the volume was normalized by Mg^2+^-pretreated nuclei, although Mg^2+^-pretreated nuclei became large after the washing (relative nuclear volume = 1), 5-H-Y-pretreated nuclei did not change (~0.1). 5-H-Y seems to bind tightly to chromatin DNA, in contrast to Mg^2+^. The error bars represent the standard deviation. **(E)** 5-H-Y induces chromatin condensation *in vivo*. HeLa cells were treated with TSA to decondense chromatin and then with 5-H-Y. 5-H-Y induced enrichment of chromatin at nuclear periphery and nucleoli (left) although we cannot exclude the possibility that condensation by 5-H-Y only occur around nucleoli and nuclear periphery. Right plot shows the intensity quantification of nuclear periphery chromatin. **p < 0.01, Chi-square test.

How does 5-H-Y inhibit DNA replication and RNA transcription? Because 5-H-Y is positively charged and induces compaction of naked DNA^26^, we examined the effects of 5-H-Y on higher-order chromatin structure. To quantitate chromatin structure in solution *in vitro*, arrays of 12 positioned nucleosomes were reconstituted from pure histones and DNA as a model chromatin (Fig. 5A, top left), followed by sedimentation velocity experiments in an analytical ultracentrifuge (Fig. 5A, bottom left). The degree of folding of the 12-mer nucleosomal arrays was described quantitatively by the sedimentation coefficient (S)^35^. The extended beads-on-a-string conformation sediments at ~29 S, whereas folding causes the nucleosomal arrays to become compact and increases the sedimentation coefficient to ~40–55 S^35^. When the nucleosomal arrays were exposed to 5-H-Y, the sedimentation coefficient increased, from 27 S to 40–55 S, in a dose-dependent manner. In contrast, cisplatin did not affect the sedimentation of the nucleosomal arrays (Fig. 5B). These results indicate that 5-H-Y, but not cisplatin, induced folding of nucleosomal arrays *in vitro*.

To further investigate nuclear chromatin folding by 5-H-Y, we used permeabilized human cell nuclei attached to glass surfaces (Fig. 5A, right)^31^. Because chromatin is negatively charged, the compaction states of nuclei and their chromatin depend on the cation concentration in the environment^31^,^36^. For example, in low cation environments (e.g., low Mg^2+^ concentration), nuclear chromatin unfolds, leading to an expansion of nuclear volume^31^. However, nuclear chromatin in the presence of a cation (e.g., 5 mM Mg^2+^) becomes highly condensed and the nuclear volume decreases^31^. As shown in Fig. 5C, nuclear volume, measured with a confocal laser scanning microscope, decreased with the addition of 5-H-Y in a concentration-dependent manner. Our results indicate that 5-H-Y can induce the folding of nuclear chromatin. Notably, permeabilized nuclei pre-treated with 5-H-Y did not increase in volume even after washing with low-salt buffer, while nuclei pre-treated with 5 mM Mg^2+^ unfolded greatly after washing (Fig. 5D). This suggests that 5-H-Y binding to nuclear chromatin DNA is quite tight and not a simple electrostatic attraction.

Next, we tested whether 5-H-Y could condense chromatin *in vivo* (Fig. 5E). To clearly visualize the chromatin condensation *in vivo*, chromatin in HeLa cells was decondensed by treatment with the HDAC inhibitor trichostatin A (TSA)^37^. When treated with 5-H-Y, the HeLa cells showed prominent chromatin condensation, especially around the nuclear periphery and nucleoli in the cells (Fig. 5E). However, control and cisplatin-treated cells showed less or no condensation. Taken together, our *in vitro* and *in vivo* tests demonstrate that tight DNA binding by 5-H-Y induces chromatin folding whereas cisplatin does not.

### DNA damage by 5-H-Y is repaired primarily by different pathways than ICL repair

The results above suggest that 5-H-Y acts on DNA differently from cisplatin. Cisplatin shows hypersensitivity in cells that are deficient in the FANC genes, the products of which are involved in ICL repair. Thus, we examined whether 5-H-Y had a different reaction in such cells. For this purpose, we took a genetics approach using chicken DT40 cells, the genes of which can be modified efficiently using homologous recombination-mediated targeting^38^.

**Figure 6:**
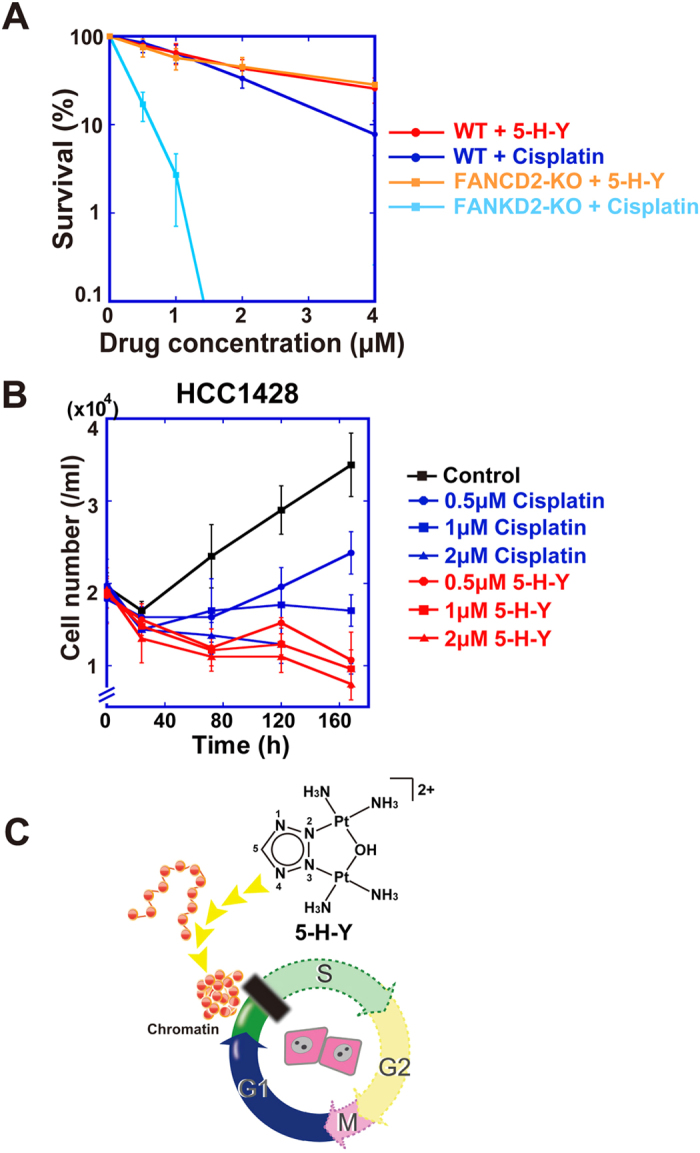
DNA damage by 5-H-Y is repaired primarily by different pathways from ICL repair. (**A**) Sensitivity assay to the 5-H-Y or cisplatin in wtDT40 cells and FUNCD2-KO cells using a colony formation assay. Mean ± SD of three independent experiments is shown. See also Fig. S7. (**B**) Cell proliferation assay of cisplatin-resistant HCC1428 cells upon 5-H-Y or cisplatin treatment. HCC1428 cells were treated with the indicated concentrations of 5-H-Y or cisplatin, and cell numbers were monitored. 5-H-Y was effective even in this cisplatin-resistant cell line. (**C**) A model figure. This study demonstrated that 5-H-Y inhibited DNA replication, and arrests the treated cells in S/G2 phase. 5-H-Y binds tightly to chromatin DNA and induces chromatin folding.

5-H-Y and cisplatin inhibited wild-type DT40 cell growth in a similar manner (Fig. S1B). Then we examined the cell viability of DT40 cells lacking one of the FANC genes, FANCD2, by colony formation assays in the presence of 5-H-Y or cisplatin^39^. FANCD2-KO cells showed no hypersensitivity to 5-H-Y while just 2 μM cisplatin was enough to completely inhibit colony formation (Fig. 6A). A similar tendency was also observed using the FANCC- and FANCJ-KO DT40 cells although they seem to be more sick and more sensitive to any perturbations than FANCD2-KO cells^40^,^41^ (Fig. S7). Taken together with the *in vitro* data, our results demonstrated that 5-H-Y has a different cytotoxic mechanism than cisplatin: DNA damage by 5-H-Y is repaired by different pathways from ICL repair.

Furthermore, 5-H-Y can be effective in cells with acquired resistance to cisplatin (Fig. 6B). Generally, tumor cells with the BRCA2 mutation show hypersensitivity to ICL-inducing agents, such as cisplatin^42^. However, such tumor cells ultimately develop cisplatin resistance^11^. For example, a BRCA2-mutated breast cancer cell line, HCC1428, partially acquired resistance to cisplatin by a secondary genetic change in BRCA2 that rescued BRCA2 function^11^. We found that HCC1428 cells still had higher sensitivity to 5-H-Y than cisplatin (Fig. 6B). Consistently, a previous report showed that 5-H-Y killed cisplatin-resistant types of PC-9 and PC-14 cells more efficiently than cisplatin^27^. These findings suggest that 5-H-Y can effectively suppress proliferation of cisplatin-resistant cancer cells.

## Discussion

Using various techniques, we demonstrated that the azolato-bridged complex 5-H-Y is incorporated into nuclei (Fig. 1) and inhibits DNA replication and RNA transcription, arresting the treated cells in the S/G2 phase (Figs 2 and 6C). 5-H-Y binds tightly to chromatin DNA and clearly induces chromatin folding *in vitro* and *in vivo* (Figs 5 and 6C). In addition, 5-H-Y has much less intra- and interstrand crosslinking ability than the commonly used anti-cancer drug cisplatin (Fig. 4). These results are consistent with genetic data that have shown that DNA damage induced by 5-H-Y is not processed by the FA/BRCA pathway, which plays an important role in the repair of cisplatin-induced ICL (Figs 6A and S7). Moreover, 5-H-Y can suppress proliferation of cisplatin-resistant cancer cells (Fig. 6B)^27^. Our study provides a mechanistic insight into the differences between 5-H-Y and cisplatin. 5-H-Y may be effective against chemotherapy-insensitive cancers, especially against platinum-refractory cancers, and could be a promising alternative to platinum-based drugs.

Regarding the inhibition mechanisms of DNA replication and RNA transcription, chromatin folding by 5-H-Y could contribute to the processes. Although the higher-order chromatin structure is not fully understood, recent evidence, including our own, suggests that interphase chromatin forms numerous condensed chromatin domains^43^,^44^, consisting of irregularly folded nucleosome fibers^36^,^45^,^46^,^47^. Because DNA replication and RNA transcription might occur at opened chromatin at the surface or outside of such compact domains^48^,^49^,^50^, we propose that 5-H-Y can inhibit the opening of chromatin and subsequent initiation processes in treated cells.

Another possibility is that the tight binding of 5-H-Y to chromatin DNA and stabilization of the DNA duplex inhibit the DNA replication and RNA transcription processes directly. Recently, one azolato-bridged complex, [{*cis*-Pt(NH_3_)_2_}_2_(μ-OH)(μ-pyrazolato)]^2+^, was shown to stay in the AT-tract minor groove of DNA by non-covalent interactions (unpublished result, Komeda *et al.*). 5-H-Y may also be a minor-groove binding agent and may act in a similar way to minor-groove binders such as netropsin and distamycin A^51^, which can stabilize the DNA duplex to suppress the unwinding of DNA, a critical first step in the DNA replication and RNA transcription.

Inhibition of DNA replication by non-covalent DNA binding could be advantageous over other cancer chemotherapy agents, because covalent modification of DNA may alter genomic information (the DNA sequence) during the DNA repair process in an irreversible way, leading to the production of abnormal proteins and also drug-induced tumorigenesis. Given that cytotoxicity by 5-H-Y is assumed to change less genome DNA sequences in non-cancer cells, genome integrity could be maintained better in such cells. Efficient PCR amplification using 5-H-Y-treated template DNA (Figs 4D and S6) supports this notion.

To date, most studies on platinum anticancer agents have focused on the binding modes and geometries of covalently bound platinum-DNA adducts: DNA intra- or interstrand crosslinks. However, it has also been suggested that non-covalent interactions between cationic platinum(II) complexes and DNA are sufficient to exhibit cytotoxic effects^52^,^53^. Accordingly, we may have found a new “pharmacophore” in non-covalent platinum-DNA interactions, with which 5-H-Y binds tightly to DNA and induces a change in chromatin structure. On the other hand, the possibility of contribution of covalent platinum-DNA adducts by 5-H-Y might also not be excluded, because 1,2-intrastrand crosslinks, probable covalent DNA adducts of one of the azolato-bridged complexes, are recognizable by DNA repair systems less efficiently than those of cisplatin^25^. Our observations of fewer and weaker γH2AX foci and a lower level of activated Chk1 in the 5-H-Y-treated cells versus cisplatin-treated cells might support this possibility.

Our study has provided a mechanistic insight into the actions of 5-H-Y, which are directly related to its effects on cisplatin-resistant cancer cells and *in vivo* antitumor efficacy against chemotherapy resistant cancers, such as pancreatic cancer. Azolato-bridged complexes are among the most promising anticancer drug candidates.

## Methods

### Chemicals

5-H-Y was prepared as reported previously^20^. Cisplatin was purchased from Bristol-Myers Squibb.

### Cell lines

Human cell lines PC9, HeLa, U2OS, and TIG-1, except HCC1428 were maintained in Dulbecco’s modified Eagle’s medium (GIBCO) supplemented with 10% (v/v) fetal bovine serum (Thermo Scientific) at 37 °C under 5% CO_2_ in air in a humidified incubator. HCC1428 cells were maintained in RPMI1640 medium supplemented with 10% FBS. Chicken DT40 cells were cultured in DMEM/high glucose medium (Sigma) supplemented with 1 mM β-mercaptoethanol (Sigma), 100 U/mL penicillin, 100 mg/mL streptomycin (GIBCO), 10% fetal calf serum (Hana-Nesco Bio), and 1% chicken serum (GIBCO) at 38.5 °C.

### Cell proliferation assay

Cells were seeded in 6-well plates (1 × 10^5^ or 2 × 10^4 ^cells/mL) with various concentrations (0–4 μM) of 5-H-Y or cisplatin. The numbers of proliferated viable cells were examined microscopically at several time points, as indicated in each figure.

### Cell viability assay

Serially diluted cells were plated in medium containing 1.5% methylcellulose. To measure sensitivity to 5-H-Y or cisplatin, exponentially growing cells were incubated in methylcellulose medium with the drugs. Colonies were counted after incubation for 1–2 weeks.

### Flow Cytometry

For flow cytometry (FCM), cells treated with 5-H-Y or cisplatin for 24 h were pulse-labeled for 60 min with 10 μM 5-ethynyl-2′-deoxyuridine (EdU). The dead cells were washed away prior to the cell harvest. After harvesting, to fluorescently label the incorporated EdU in newly synthesized DNA, we used Click-iT EdU Flow Cytometry Assay kits (Invitrogen). The cells were also stained with FxCycle Far Red Stain (Invitrogen) to stain DNA. FCM analysis was performed with a JSAN cell sorter (Bay Bioscience) using a logarithmic FL1-A channel for EdU detection and a linear FL5-A setting for FxCycle Far Red Stain. The cells with abnormal shapes or multiple nuclei were eliminated by forward/sideward scatter (FSC/SSC) gating. Analysis was performed using the Flowlogic software. For each analysis, we started with ~10^6 ^cells and ~10^4 ^cells of the flow cytometer result were plotted.

### *In vivo* measurement of RNA transcription

HeLa cells on coverslips were cultured with 2 μM of 5-H-Y or cisplatin for 24 h and in the last 1 h with 50 μM of 5-ethynyl uridine (EU). The cells were then treated with 3.7% formaldehyde and then with 0.5% triton X-100 for permeabilization. Incorporated EU was fluorescently labeled by Click-iT reaction (Invitrogen) using Click-iT Alexa Fluor 594 dye. DNA was then stained with 0.5 μg/ml DAPI. The mounted cells were observed under a DeltaVision microscope (Applied Precision) and analyzed using the ImageJ software (NIH, Bethesda, MD, USA)^56^.

### Immunofluorescence staining and immunoblotting

Immunofluorescence staining was performed as described previously ref. 65. The primary antibody, anti-phospho H2AX (Ser139) mouse monoclonal (Upstate), and the secondary antibody, Alexa-Fluor-594-conjugated goat anti-mouse IgG (Invitrogen), were used at dilutions of 1:3000 and 1:1000, respectively. The samples were analyzed under a DeltaVision microscope (Applied Precision). Images were analyzed using the ImageJ software^56^.

For immunoblotting, the following antibodies were used at the indicated dilutions: anti-phospho Chk1 (Ser345) rabbit polyclonal (Cell Signaling Technology, Inc.) at 1:500, anti-Chk1 mouse monoclonal (MBL) at 1:1000, anti-histone H2B rabbit polyclonal (upstate) at 1:10000, horseradish peroxidase-linked anti-rabbit IgG whole antibody (Bio-Rad) at 1:5000 for anti-phospho Chk1 and at 1:30000 for anti-histone H2B, and horseradish peroxidase-linked anti-mouse IgG whole antibody (Bio-Rad) at 1:5000. Cells were lysed in FSB buffer. After denaturation at 95 °C for 5 min, proteins in lysates were separated using SDS-PAGE. Proteins were transferred to an Immobilon-P membrane (Millipore) and blotted with antibodies after blocking in PBS-T containing 5% BSA for staining phospho-Chk1 and 3% skim milk for staining Chk1and H2B for 30 min at RT. Detection was performed using the Immobilon Western Chemiluminescent HRP substrates (Millipore) with EZ-Capture MG (ATTO).

### Sedimentation Velocity of Nucleosomal Arrays and 5-H-Y

Nucleosomal arrays were assembled as described^57^, using a 12-mer 601^58^ DNA template and native chicken core histone octamers^59^. Samples were prepared for the analytical ultracentrifuge by diluting to an absorbance of approximately 0.6 at 260 nm, and the 5-H-Y or Cisplatin added to the appropriate concentration.

Sedimentation velocity experiments were conducted in a Beckman XL-A/I analytical ultracentrifuge at 17,000 RPM using absorbance optics as described^60^. The scans were analyzed using the enhanced van Holde-Weischet method^61^ implemented in the Ultrascan II data analysis software^62^ to yield an integral distribution of diffusion-corrected sedimentation coefficients.

### Chromatin compaction assay by measurement of nuclear volume

For condensed chromatin, isolated nuclei (~1 × 10^7^) were suspended in HM buffer (10 mM HEPES-KOH, pH 7.4, and 5 mM MgCl_2_) and attached to poly L-lysine-coated coverslips by centrifugation (400 × *g*, 5 min)^31^. For decondensed chromatin, the nuclei on the coverslips were gently transferred to HM buffer or 1 mM EDTA buffer (pH 8.0). The nuclei were treated with 5-H-Y overnight at room temperature in the dark. Hereinafter, all solutions included 5-H-Y. After fixation with 1% formaldehyde, the nuclei were washed with 50 mM glycine and stained with 2 μM TO-PRO-3 solution (Invitrogen) at 37 °C for 30 min. After washing, *z*-stack images were acquired using an LSM510 META laser scanning confocal microscope (Carl Zeiss, Wetzlar, Germany) with a 100 × objective at 0.48 μm intervals. The images were processed using the LSM Image Browser (Carl Zeiss) and ImageJ software^56^.

To examine whether buffer washing removed 5-H-Y from chromatin, 5-H-Y-pretreated chromatin or 5 mM Mg^2+^-pretreated chromatin were further washed with 1 mM EDTA without 5-H-Y three times. Then, nuclear volumes were measured as described above and normalized by the average volume of 5 mM Mg^2+^-pretreated nuclei.

### Observation of chromatin compaction *in vivo*

At first, we could not see notable effect on chromatin in the 5-H-Y-treated cells, presumably because of the resolution limitation by a conventional light microscopy. To enhance a possible effect, the cells were first treated with 500 nM trichostatin A (TSA) for 4 h to decondense chromatin in the cells (e.g. Ref. 37). Then 10 μM 5-H-Y or cisplatin was added to the TSA-treated cells and further incubated for 1 h. The cells were observed by live cell imaging with a fluorescent microscope (Nikon Eclipse Ti2000-E). We used oblique illumination microscopy^63^,^64^. For the quantification of condensation, fluorescent intensity of the nuclear rim (average width of 5 pixels, 320 nm) and the nucleoplasm (average width of 10 pixels, 640 nm) were measured by line scan method. The induced condensation was evaluated by the ratio of nuclear rim intensity to nucleoplasm intensity. These analyses were performed with the ImageJ software^56^. The statistical significance was evaluated by Chi-square test.

### PCR using drug-treated plasmid DNA

pUC19 and pBluescript II were linearized by EcoRI digestion, recovered by ethanol precipitation, and treated with 2.5 μM cisplatin or 5-H-Y (DNA base:drug = 30:1) for 24 h or 48 h at 37 °C. After purification using Wizard SV Gel and the PCR Clean-Up System (Promega), 50 ng purified plasmid was used as templates for PCR with the following set of primers: primer F, AGCAAAAACAGGAAGGCAAA and primer R, ACTGGCCGTCGTTTTAC. PCR was performed with the KOD-Plus kit (Toyobo) according to the manufacturer’s protocol. The cycle numbers used were 4, 7, and 10 cycles. The PCR products were electrophoresed on 0.8% agarose gels and stained with EtBr to visualize DNA.

### Alkaline agarose electrophoresis

To detect interstrand crosslinks in drug-treated DNAs, alkaline agarose gel electrophoresis was carried out. pUC19 and pBluescript II were linearized by EcoRI digestion, recovered by ethanol precipitation, and treated with 2.5 μM cisplatin or 5-H-Y (DNA base:drug = 30:1) for 24 h or 48 h at 37 °C. After purification using Wizard SV Gel and the PCR Clean-Up System (Promega), 1 μg of each purified plasmid was mixed in a buffer containing 50 mM NaOH, 1 mM EDTA (pH 8.0), 3% (w/v) Ficoll, and 0.0425% (w/v) xylene cyanol. The plasmid samples were electrophoresed on 0.8% alkaline agarose gel in 50 mM NaOH and 1 mM EDTA (pH 8.0). After electrophoresis, the gel was neutralized in a buffer containing 1 M Tris-HCl (pH 7.6) and 1.5 M NaCl for 45 min and stained with 0.3 μg/mL EtBr in 1 × TAE buffer (40 mM Tris, 20 mM sodium acetate, and 1 mM EDTA, pH 8.0).

## Additional Information

**How to cite this article**: Imai, R. *et al.* Chromatin folding and DNA replication inhibition mediated by a highly antitumor-active tetrazolato-bridged dinuclear platinum(II) complex. *Sci. Rep.* **6**, 24712; doi: 10.1038/srep24712 (2016).

## Supplementary Material

Supplementary Information
