# Supplementary material for: Chromatin folding and DNA replication inhibition mediated by a highly antitumor-active tetrazolato-bridged dinuclear platinum(II) complex
Source: Sci Rep. 2016 Apr 20;6:24712. doi: 10.1038/srep24712 (PMC4837362; doi:10.1038/srep24712)
Supplement: Supplementary Information [file srep24712-s1.pdf]

Supplementary Information

Chromatin folding and DNA replication inhibition mediated by a highly antitumor-active tetrazolato-bridged dinuclear platinum(II) complex

Ryosuke Imai[1,2], Seiji Komeda[3], Mari Shimura[4,5,6], Sachiko Tamura[1,4], Satoshi Matsuyama[4,6,7], Kohei Nishimura[8], Ryan Rogge[9], Akihiro Matsunaga[4,5,6], Ichiro Hiratani[1,2], Hideaki Takata[1,10], Masako Uemura[3], Yutaka Iida[11], Yuko Yoshikawa[12], Jeffrey C. Hansen[9], Kazuto Yamauchi[4,6,7], Masato T. Kanemaki[2,8,13], Kazuhiro Maeshima[1,2,4,6*]

Supplementary Methods

Scanning X-ray fluorescence microscopy (SXFM)

SXFM was set up at an undulator beamline, BL29XU, of the SPring-8 synchrotron radiation facility in Japan by combining a Kirkpatrick-Baez type X-ray focusing system [54,55], an *xy*-scanning stage for sample mounting, and an energy-dispersive X-ray detector (Vortex-90EX, Hitachi High-Technologies Science America, Inc.). For element array analysis, monochromatic X-rays at 15 keV for Pt *L*-line excitation were focused into a 1000 nm (H) × 1000 nm (W) spot with a measured flux of $\sim 3\times10^{11}$ photons/s. The focused X-rays simultaneously yielded the fluorescence of various chemical species in a small volume of sample cells. The X-ray fluorescence spectrum was recorded with an exposure of 4–10 s at each pixel of scanning. The fluorescence signals of each element of interest were extracted and normalized by incident beam intensity. After scanning the whole area, elemental distributions were visualized digitally. In addition to the mapping images, an elemental concentration was analyzed quantitatively using thin platinum films, of which the thickness and the density were determined in advance. PC9 cells were plated on acrylic-based prolene film with carbon deposition (Jeol, Tokyo, Japan), which were washed three times with 70% EtOH (Wako Pure Chemical, Osaka Japan). After cells had adhered well, platinum compounds were treated for 24 h. After fixation with 2% paraformaldehyde in phosphate-buffered saline (PBS) for 10 min, cells were

washed once in PBS prior to washing with 70% EtOH and dried. Note that we washed out dead cells and fixed only living cells.

Measurement of cellular platinum by ICP-MS

PC9 cells were harvested 24 h after the addition of cisplatin or 5-H-Y. We washed out dead cells and harvested only living cells. To purify whole cells, we centrifuged the cell suspension and collected the cells. To obtain the nuclear fraction, cells were suspended in HBSS buffer containing 0.2 mM phenylmethylsulphonyl fluoride (PMSF) and 0.1% Triton X-100. Then the nuclei were collected by centrifugation. To isolate the DNA fraction, we performed the following procedure. We treated whole cells with SDS to lyse the cells, and then the lysate was treated with proteinase K (WAKO). DNA was isolated from the lysate by phenol-chloroform precipitation and ethanol precipitation, then redissolved in MilliQ water. Then the solution was treated with 0.04 mg/mL RNaseA. The DNA was purified by phenol-chloroform precipitation and ethanol precipitation again and finally redissolved in TE buffer (pH 8.0). Each fraction was snap-frozen in liquid nitrogen. As blanks, we performed all of the fractionation steps in the absence of any cells. To determine the metal concentration of platinum in whole cells or cellular fractions, about ~5×10^6 cells were wet-digested with 1.0 mL HNO_3 at 160°C for 12 h. Concentrations of ^{194}Pt and ^{195}Pt were determined by the ICP-MS

(ELAN DRC II, Perkin Elmer, MA, USA).

Quantitation of covalent Pt-DNA adducts

Double-helical calf-thymus (CT) DNA solutions (100 μM, relative to the monomeric nucleotide content) were incubated with 8 μM cisplatin or 5-H-Y in a buffer solution composed of 10 mM HEPES (pH 7.5), 100 mM $KClO_4$, and 2 mM $MgCl_2$ at 37°C. At various time intervals, an aliquot (500 μL) of the reaction mixture was withdrawn, and 100 μL 4 M NH_4Cl was added to each reaction mixture immediately after sample collection to prevent further platination. Then each sample solution was poured into a cation exchange SPE tube (Discovery DSC-WCX SPE Tube, SUPELCO), which was washed with 250 μL MilliQ water twice and then multiple steps of ultrafiltration (1,000 *g*, 4°C, 6–7 min) were performed to remove non-covalently bound platinum complexes from the eluate, using a centrifugal ultrafiltration device (Nanosep and Nanosep MF Centrifugal Devices, Pall) after adding the following solutions: 250 μL 4 M NH_4Cl (three times), 25 μL 4 mM EDTA, 25 μL 4 M NaOH and MilliQ water to a final volume of 500 μL including DNA solution (once), 250 μL 4 M NH_4Cl (three times), and 250 μL MilliQ water (three times). After ultrafiltration, the total monomeric nucleotide content of covalently platinated CT DNA in each sample solution was determined by measuring UV absorbance at 260 nm using a spectrophotometer (GeneQuant, GE Healthcare). Then each DNA solution was sonicated, ashed in

concentrated HNO_3, and redissolved in 2% HNO_3 for ICP-MS measurements (Agilent 7500cs, Agilent Technology) to determine the platinum content in the DNA. The R_b value, defined as the molar ratio of platinum complex bound per nucleotide, was calculated from the DNA and platinum content in each sample.

In vivo EdU labeling

Cells were seeded on poly-L-lysine-coated coverslips. The cells were treated with 10μM of 5-H-Y or cisplatin for 1 h. 5 μM EdU was added to the drug-treated cells and further incubated for 1 h. The cells were then fixed with 1.85% formaldehyde and permeabilized with 0.5% triton X-100. Incorporated EdU was labeled by Click-iT reaction (Invitrogen) using EdU Alexa Fluor 594 dye. DNA was stained with 0.5 μg/ml DAPI. The mounted cells were observed under a DeltaVision microscope (Applied Precision) and analyzed using the ImageJ software [56].

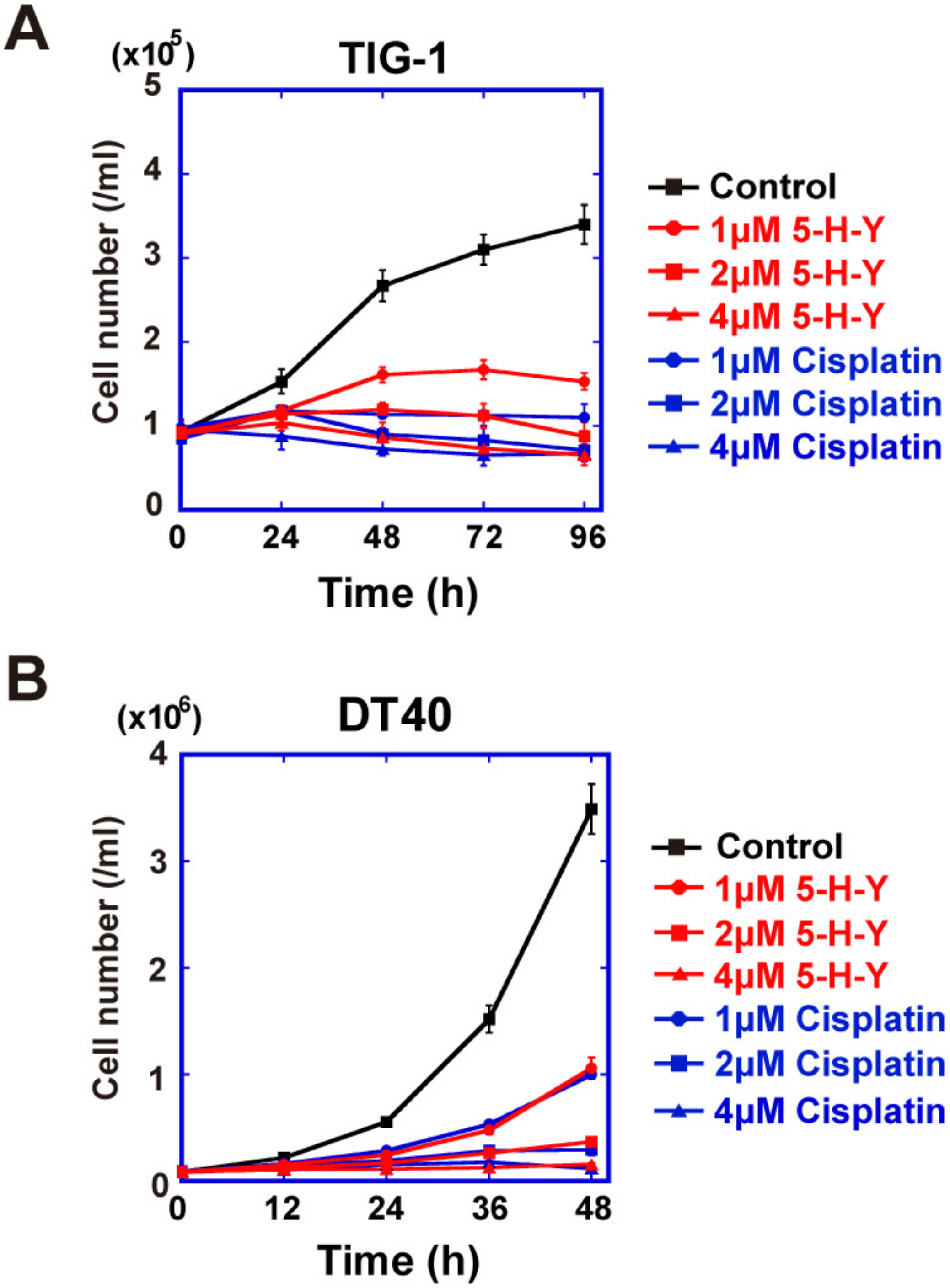

Supplementary Figure S1

Cell proliferation of human normal fibroblast TIG-1 cells **(A)** and chicken DT40 cells **(B)** upon 5-H-Y or cisplatin treatment. The cell numbers were monitored from 0 to 96 h for TIG-1**(A)** and from 0 to 48 h for DT40 cells **(B)**.

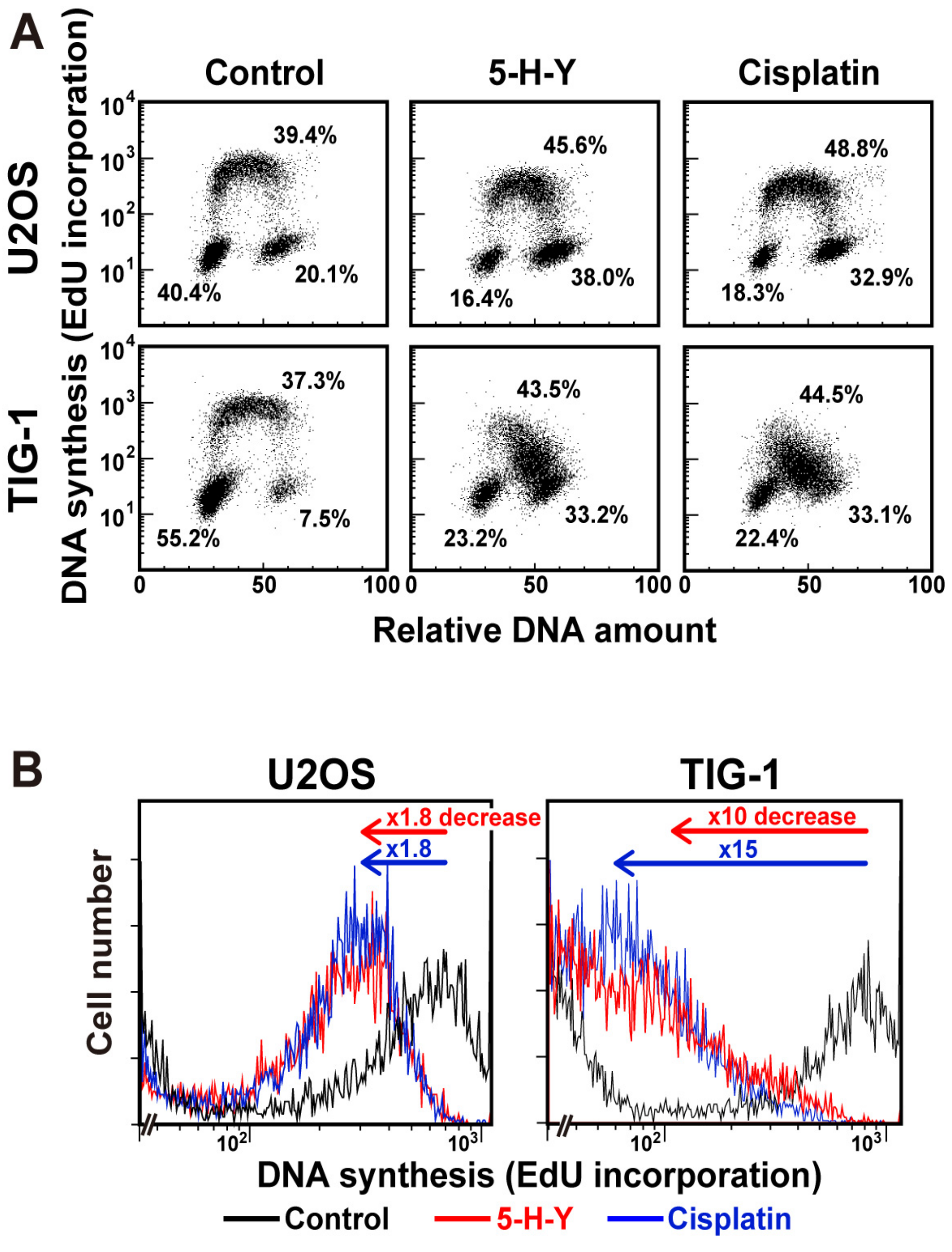

Supplementary Figure S2

5-H-Y inhibits DNA replication of U2OS and TIG-1 cells.

(A) Flow cytometry results for U2OS cells (upper row) and TIG-1 cells (lower row) with/without 5-H-Y (2 µM) or cisplatin (2 µM). Percentages of each cell cycle population are indicated. **(B)** DNA synthesis (EdU incorporation) versus cell numbers plots of (A). Fold-decreases in EdU incorporation upon 5-H-Y (red) or cisplatin (blue) treatment are indicated. Note the several-fold decrease in EdU incorporation in the 5-H-Y (red) and cisplatin (blue) treated cells.

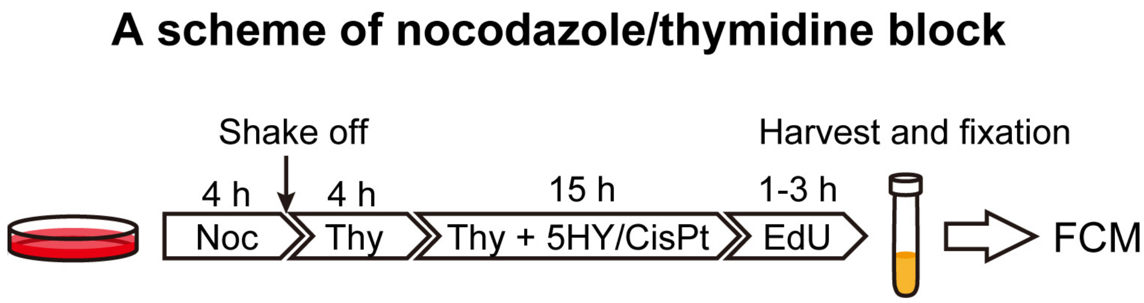

Supplementary Figure S3

Schematic of nocodazole-thymidine block to collect G1/S cells.

Unsynchronized HeLa cells were treated with 80 ng/mL nocodazole for 4 h. After synchronization at the mitotic phase, mitotic cells were isolated by shaking off and were incubated in medium containing 2.5 mM thymidine to block the cells in the G1/S phase. Then, 4 h later, 2 μM 5-H-Y or cisplatin was added to thymidine-containing medium and the cells were incubated for a further 15 h. Then the synchronized cells were released from the thymidine block and labeled with 10 μM EdU for 1or 3 h (see also Fig. S3). Harvesting and staining for FCM were the same as for unsynchronized cells.

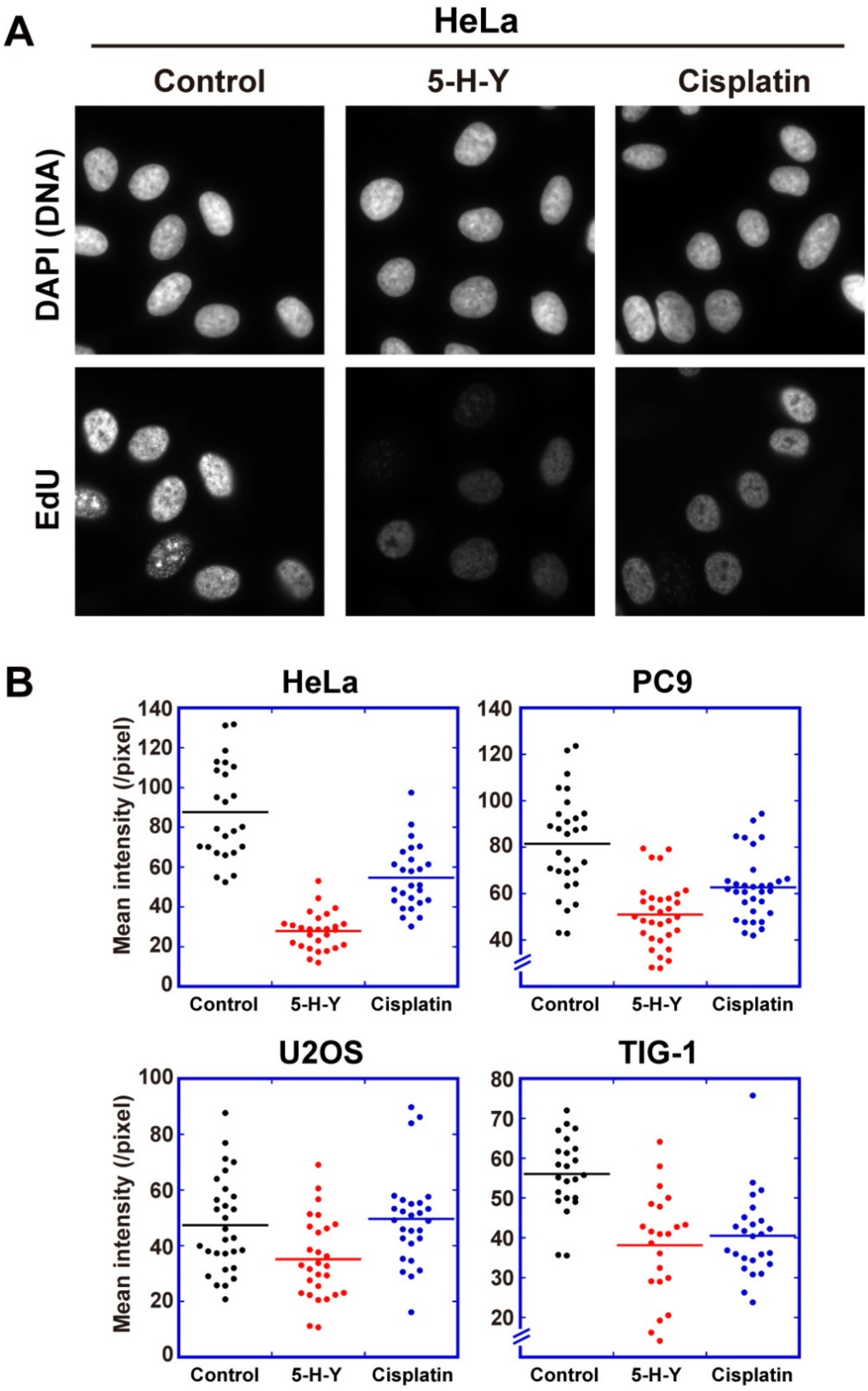

Supplementary Figure S4

Effect of short-time treatment of 5-H-Y on DNA replication in the four human cell lines. (A) DNA replication foci in HeLa cell nuclei were labeled with EdU and observed by fluorescent microscope. In a short time, 5-H-Y is more effective to inhibit DNA replication than cisplatin. **(B)** Average intensities in each cell were quantified and plotted (lower) (each group, N >20).

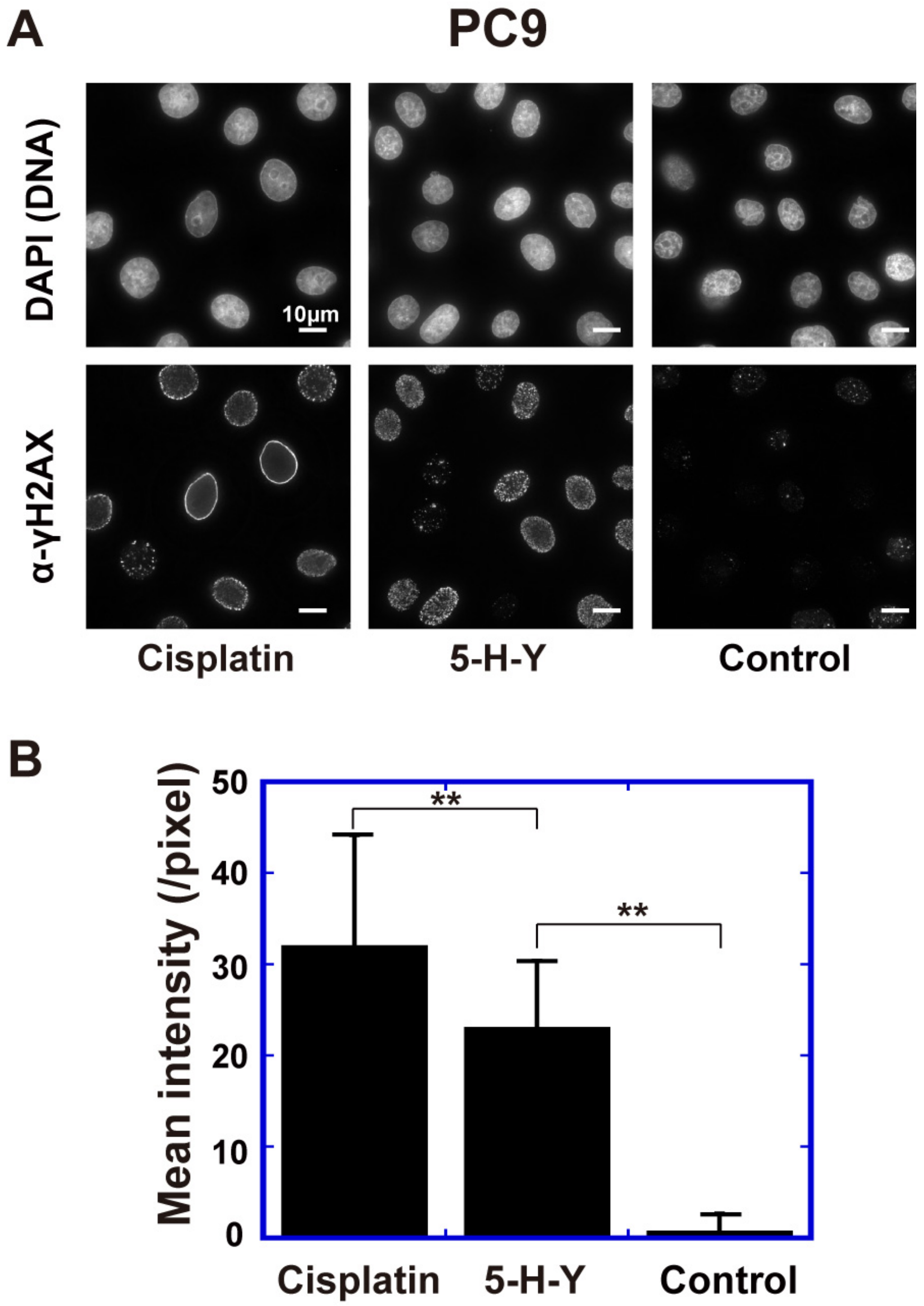

Supplementary Figure S5

γH2AX foci formation in 5-H-Y- or cisplatin-treated PC9 cells.

(A) DNA stain, upper; anti-γH2AX antibody staining, lower. Scale bars show 10 μm. The signal in 5-H-Y-treated cells was localized mainly in the nuclear periphery, which seemed to be different from the signal localization in cisplatin-treated cells. **(B)** Quantification of the γH2AX signal intensity averaged from ~50 nuclei. **$p < 0.01$, Student's t-test.

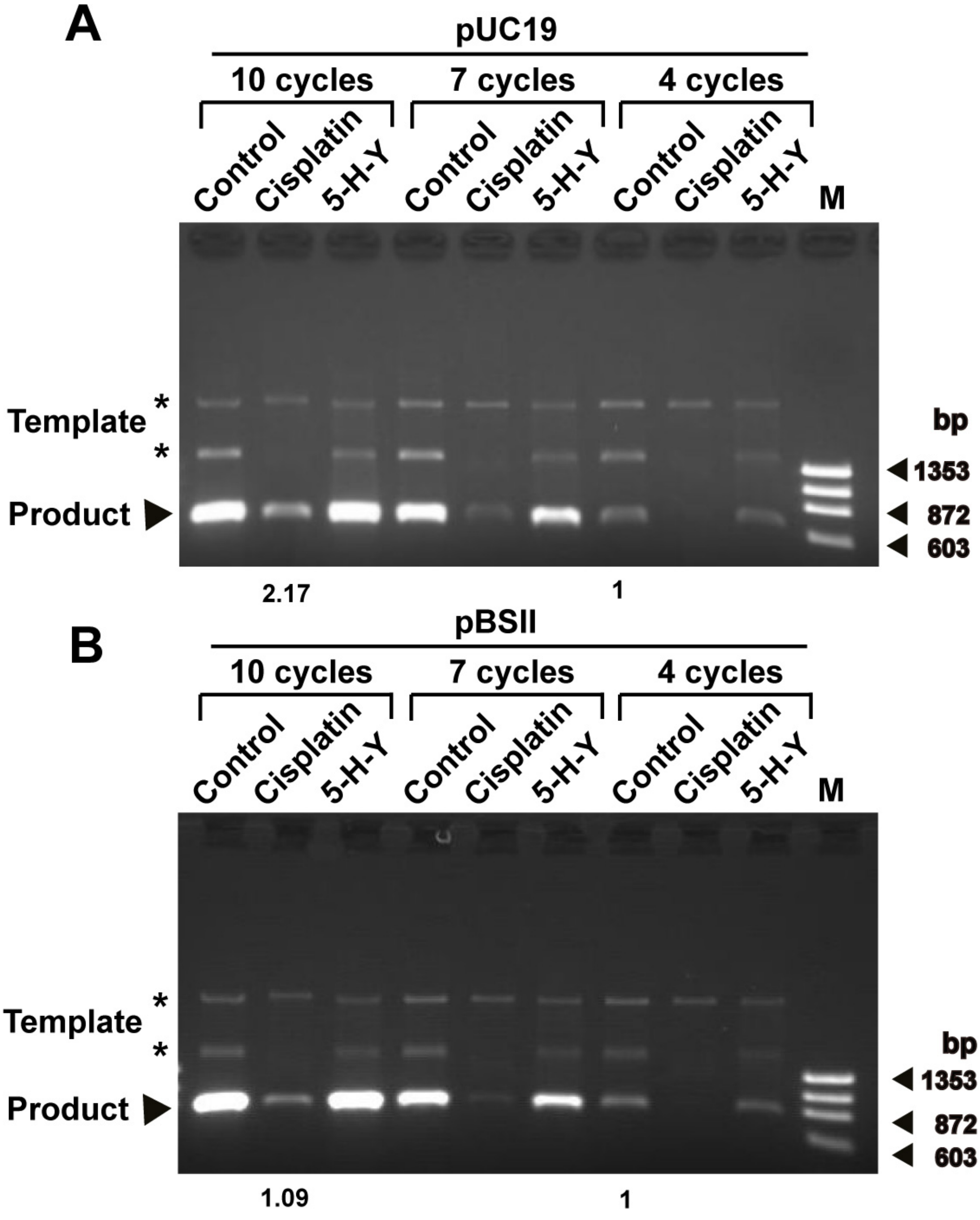

Supplementary Figure S6

Semi-quantitative PCR with drug-treated templates.

PCR reactions were performed with various cycle numbers (10, 7, or 4 cycles) for no drug (Control), cisplatin-, or 5-H-Y-treated pUC19 **(A)** or pBluescript II (pBSII) **(B)**. The PCR products (marked with arrow) on the agarose gel after electrophoresis are shown. Note that the amount of product for the PCR at 10 cycles using cisplatin-treated template is similar to the control product at 4 cycles.

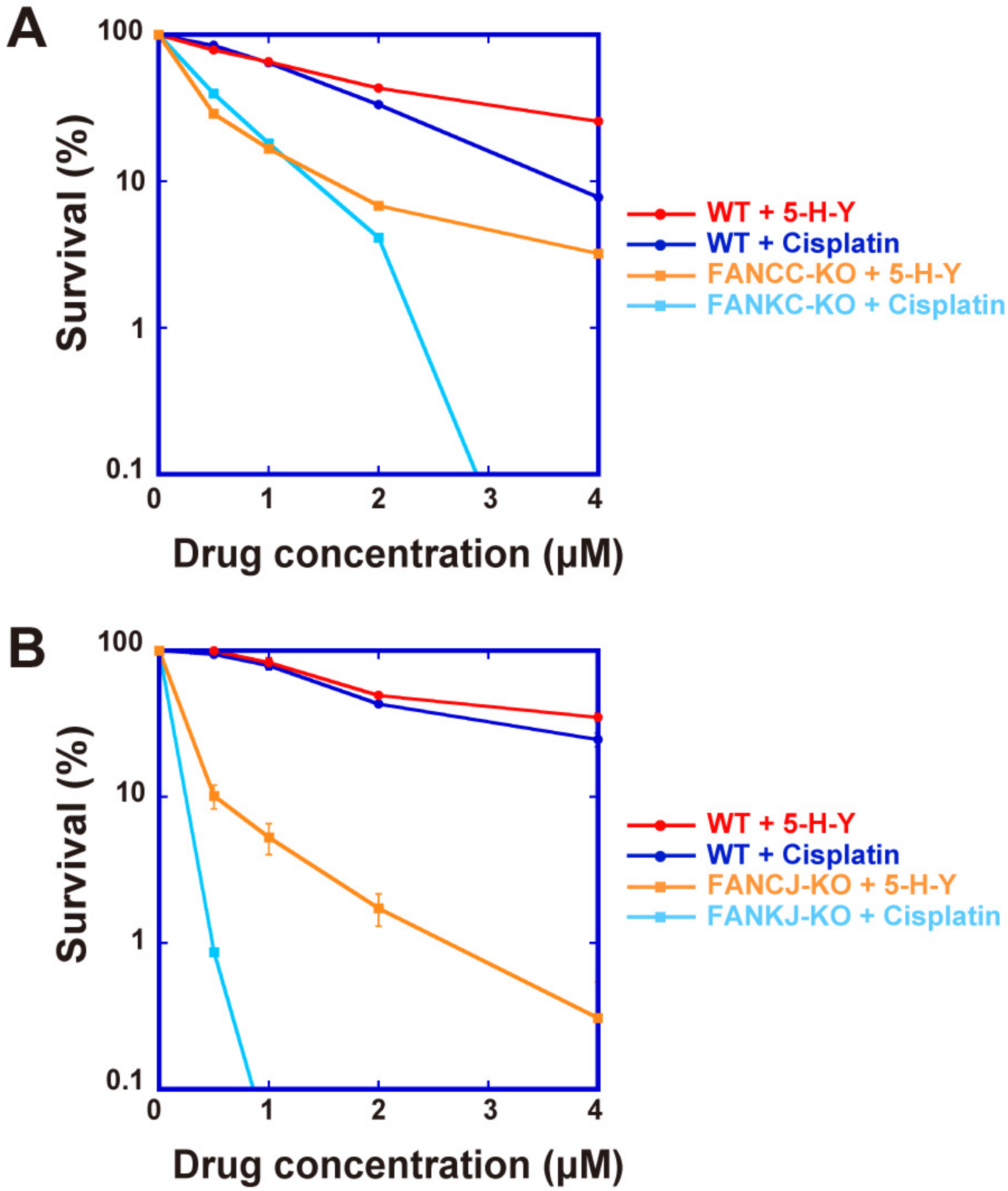

Supplementary Figure S7

Sensitivity assay to the 5-H-Y or cisplatin in several cell lines.

Sensitivity assays of FANCC-KO DT40 **(A)** and FANCJ-KO DT40 **(B)** tested by colony formation assay. Mean ± SD of three independent experiments is shown.
